# Merkel Cells Release Glutamate Following Mechanical Stimulation: Implication of Glutamate in the Merkel Cell-Neurite Complex

**DOI:** 10.3389/fncel.2019.00255

**Published:** 2019-06-11

**Authors:** Asuka Higashikawa, Maki Kimura, Miyuki Shimada, Sadao Ohyama, Wataru Ofusa, Masakazu Tazaki, Yoshiyuki Shibukawa

**Affiliations:** Department of Physiology, Tokyo Dental College, Tokyo, Japan

**Keywords:** Merkel cell, glutamate, mechano-sensory transduction, synaptic transmission, trigeminal ganglion neuron

## Abstract

Merkel cells (MCs) have been proposed to form a part of the MC-neurite complex with sensory neurons through synaptic contact. However, the detailed mechanisms for intercellular communication between MCs and neurons have yet to be clarified. The present study examined the increases in intracellular free Ca^2+^ concentration ([Ca^2+^]_i_) induced by direct mechanical stimulation of MCs. We also measured [Ca^2+^]_i_ in the trigeminal ganglion neurons (TGs) following direct mechanical stimulation to the MCs in an MC-TGs coculture. The MCs were isolated from hamster buccal mucosa, while TGs were isolated from neonatal Wistar rats. Both cell populations showed depolarization-induced [Ca^2+^]_i_. Direct mechanical stimulation to MCs increased [Ca^2+^]_i_, showing stimulation strength dependence. In the MC-TGs coculture, the application of direct mechanical stimulation to MCs resulted in increased [Ca^2+^]_i_ in the TGs. These changes were significantly suppressed by antagonists of glutamate-permeable anion channels (4,4′-diisothiocyanato-2,2′-stilbenedisulfonic acid; DIDS), and non-competitive antagonist of the *N*-methyl-*D*-aspartate (NMDA) receptors (MK801). Apyrase, an ATP-degrading enzyme, and suramin, a non-selective P2 purinergic receptor antagonist, did not exert inhibitory effects on these [Ca^2+^]_i_ increases in the TGs following MC stimulation. These results indicated that MCs are capable of releasing glutamate, but not ATP, in response to cellular deformation by direct mechanical stimulation. The released glutamate activates the NMDA receptors on TGs. We suggest that MCs act as mechanoelectrical transducers and establish synaptic transmission with neurons, through the MC-neurite complex, to mediate mechanosensory transduction.

## Introduction

Merkel cells (MCs) have been proposed to form a part of the MC-neurite complex by establishing synaptic contact with primary sensory afferents of myelinated Aβ neurons (Halata et al., 2003). The MC-neurite complexes act as slowly adapting type I mechanoreceptors, which produce pressure sensation in both the skin and mucosa. The MCs are frequently discovered in touch-sensitive areas of the glabrous epidermis, including the outer root sheaths of hair follicles and the oral mucosa (Boulais and Misery, 2007). In the oral mucosa, MCs are located at the basal layer of the stratified squamous epithelium.

We have previously reported that MCs are capable of detecting cellular deformation by hypotonic stimulation via the activation of the transient receptor potential (TRP) cation channel subfamily vanilloid (V) members 1, 2, and 4, as well as ankyrin (A) subfamily member 1 (Soya et al., 2014). Most recently, the two-receptor-site model has been demonstrated (Woo et al., 2014), in which both MCs and innervating slowly adapting type I sensory afferents fulfill together a mechanosensory role; Piezo2 channels are essential MC mechanical transducers to signal static stimuli (i.e., pressure) (Woo et al., 2014), while slowly adapting type I sensory afferents transduce dynamic stimuli (Maksimovic et al., 2014). Deformations of the skin initiate action potential firing in slowly adapting type I sensory afferents through the activation of mechanotransduction channels at the onset of stimuli. Simultaneously, the deformation activates Piezo2 mechanosensitive channels in MCs. The activation of Piezo2 channels elicit depolarization of these cells, and induce Ca^2+^ influx via voltage-activated calcium channels (Maksimovic et al., 2014). Although the Ca^2+^ entry is thought to release neurotransmitters to the sensory afferent that generates sustained action potential firing, the substrate of neurotransmitter(s) to drive neural communication between MCs and neurons has yet to be elucidated.

The MCs consist of dense-core granules that contain a variety of neuropeptides, such as serotonin, met-enkephalin, chromogranin A, calcitonin gene-related peptide (CGRP), and vasoactive intestinal peptide (Hartschuh et al., 1989). In addition, ATP or glutamate is also reported to be a candidate neurotransmitter between MCs and neurons (Nakatani et al., 2014).

To elucidate the functional properties of neurotransmission in the MC-neurite complexes, and to determine the neurotransmitters that are released from mechanically stimulated MCs to the associated nerve endings, we investigated the changes in intracellular free Ca^2+^ concentration ([Ca^2+^]_i_) in the trigeminal ganglion neurons (TGs) following direct mechanical stimulation to MCs in an MC-TGs coculture system.

## Materials and Methods

### Ethics Statement

This study was carried out in accordance with the recommendations of the Guiding Principles for the Care and Use of Animals in the Field of Physiological Sciences approved by the Council of the Physiological Society of Japan and the American Physiological Society. This study was also carried out in accordance with the Treatment of Experimental Animals at Tokyo Dental College, and the recommendations of the National Institute of Health in the United States regarding the care and use of animals for experimental procedures, as well as the UK Animals (Scientific Procedures) Act 1986. The protocol was approved by the Ethics Committee for the Treatment of Experimental Animals at Tokyo Dental College (Approval Nos. 260301, 260304, 270303, 280303, and 290305).

### Isolation of Epithelial Cells Including MCs From the Hamster Buccal Mucosa

Epithelial cells including MCs were isolated from the bulge on the epithelium in the mucosa inside the buccal pouch, named the touch dome, of male Syrian golden hamsters (3–5 weeks old) (Soya et al., 2014). To identify single MCs after isolation, a standard-extracellular solution (ECS) containing quinacrine dihydrochloride, an MC-specific marker (Crowe and Whitear, 1978; Yamashita et al., 1992; Ikeda et al., 1994), was intraperitoneally (*i.p.*) injected (15 mg/kg) 12 h prior to the MC isolation procedure. For the isolation, Syrian golden hamsters were deeply anesthetized with sodium pentobarbitone (30 mg/kg *i.p*.; Kyoritsuseiyaku, Tokyo, Japan), following administration of isoflurane (2.0 Vol%). The buccal mucosa including the touch domes were excised and treated using the standard-ECS containing dl-dithiothreitol (3 mg/mL) at 37°C for 10 min to separate the stratified squamous epithelium from the buccal mucosa. Note that following an MC-specific marker *i.p.* injection, the animals had survived before, but were sacrificed after excision of the buccal mucosa including the touch domes. The separated epithelium was treated enzymatically with Ca^2+^ free standard-ECS containing 0.025% collagenase and 0.01% trypsin at 37°C for 10 min, and rinsed with fresh standard-ECS. The enzymatically treated stratified squamous epithelium was rubbed carefully together in the standard-ECS for mechanical dissociation in order to obtain single epithelial cells including MCs. The suspended epithelial cells in standard-ECS were triturated carefully and plated onto poly-L-lysine-coated dishes (CORNING, Corning, NY, United States) and subsequently incubated and maintained at 37°C and 5% CO_2_ for 2 h before cell identification. To establish the MC-TGs coculture system and to identify the two dimensional location of quinacrine-positive MCs on the culture dish, we manually placed marks on the bottom of the dish using a 29G needle (Terumo, Tokyo, Japan) under the fluorescence microscope and recorded its orientation and coordinate against the stage of the microscope. Quinacrine-fluorescence was measured at 525 nm in response to an excitation wavelength of 427 nm using a fluorescence microscope (IX71, Olympus, Tokyo, Japan) and the HCImage system (Hamamatsu Photonics, Shizuoka, Japan).

### Isolation of TGs

The TGs were dissected and isolated from male and female neonatal Wistar rats (6–9 days old) from the base of the brain under pentobarbital sodium anesthesia (30 mg/kg, *i.p.*) following the administration of isoflurane (2.0 Vol%). Cells were dissociated by enzymatic treatment with Hank’s balanced salt solution (Life Technologies, Carlsbad, CA, United States) containing 20 U/mL papain (Worthington, Lakewood, NJ, United States) for 20 min at 37°C while shaking (160 rpm). The dissociated TGs were resuspended and triturated in Leibovitz’s L-15 medium (Life Technologies) containing 10% fetal bovine serum, 1% penicillin-streptomycin, 1% fungizone, 26 mM NaHCO_3_, and 30 mM glucose (pH 7.4).

### Preparing MC-TGs Cocultures

Two hours after the identification of quinacrine-positive MCs on the culture dish, standard ECS was removed and the suspension including acutely isolated TGs in Leibovitz’s L-15 medium were immediately added to a dish of acutely isolated epithelial cells including MCs. The coculture was incubated in Leibovitz’s L-15 medium at 37°C for 60–120 min before loading fura-2 (see below).

### Measurements of Fura-2, Ca^2+^-Sensitive Dye Fluorescence

For the measurements of Ca^2+^-sensitive dye fluorescence, the cells in the MC-TGs coculture were also incubated in 10 μM fura-2-AM (Dojindo laboratories, Kumamoto, Japan) containing 0.1% (w/v) pluronic acid F-127 (Life Technologies) in standard ECS at 37°C for 45 min, followed by rinsing with standard ECS. A dish with fura-2-loaded cells in the coculture was mounted on the stage of a microscope (IX71) with the HCImage system (Hamamatsu Photonics). A culture dish with fura-2-loaded epithelial cells and TGs was placed again on the stage of a microscope by aligning its orientation and coordinates with those of the previously recorded orientation. Based on the marks that were previously placed on the culture dish, we confirmed the quinacrine-positive cells. We measured the fura-2 fluorescence simultaneously from the quinacrine-positive MCs and TGs located near mechanically stimulated MCs (see below). The microscope was equipped with an excitation wavelength selector and an intensified charge-coupled device camera system (Hamamatu Photonics). Fura-2 fluorescence emission was measured at 510 nm in response to alternating excitation wavelengths of 340 nm (F340) and 380 nm (F380). The [Ca^2+^]_i_ was measured as the fluorescence ratio (R_F340/F380_). We normalized the R_F340/__F380_ value (*F*) with the resting value (*F*_0_), and expressed as *F/F*_0_ units. To avoid the formation of any intercellular electrical/physical contact, we measured [Ca^2+^]_i_ in the cells within 2–5 h after preparing the MC-TGs cocultures. All experiments were conducted at room temperature (30 ± 1.0°C).

### Direct Mechanical Stimulation of a Single MC

Direct mechanical stimulation (Sato et al., 2015; Shibukawa et al., 2015; Nishiyama et al., 2016) was applied using a fire-polished glass micropipette with a tip diameter of 2–3 μm. The stimulation micropipettes were pulled from capillary glass (Harvard apparatus, Holliston, MA, United States) by using a DMZ Universal Puller (Zeitz instruments, Martinsried, Germany), were filled with standard ECS, and operated using a micromanipulator (MHW-3, Narishige, Tokyo, Japan). The micropipette was placed at a site immediately just above the cell attached position and was gently moved by 4.3, 8.5, or 12.8 μm in the vertically downward direction at a 2.8-μm/s velocity to depress the cell membrane and generate a focal mechanical stimulation. The stimulation was applied for 5 s, after which the pipette was retracted at the same velocity. The stimuli were applied no more than three times to avoid unfavorable cell damage. For the [Ca^2+^]_i_ measurement following the MC mechanical stimulation, we chose the neighboring TGs that had no close contact with MCs or neighboring TGs.

### Solutions and Reagents

Standard ECS consisted of the followings: 136 mM NaCl, 5 mM KCl, 2.5 mM CaCl_2_, 0.5 mM MgCl_2_, 1.2 mM NaH_2_PO_4_, 11 mM glucose, and 12 mM NaHCO_3_ (328 mOsm/L). To obtain high-KCl solutions, we increased the extracellular K^+^ in the standard ECS to 100 mM and reduced extracellular Na^+^ in to 41 mM. The Hanks’ balanced salt solution consisted of the following ingredients: 137 mM NaCl, 5.0 mM KCl, 2.0 mM CaCl_2_, 0.5 mM MgCl_2_, 0.44 mM KH_2_PO_4_, 0.34 mM Na_2_HPO_4_, 4.17 mM NaHCO_3_, and 5.55 mM glucose. The pH of these solutions was adjusted to 7.4 using Tris (Wako Pure Chemicals, Osaka, Japan). Suramin and MK801 were obtained from Tocris Bioscience (Ellisville, MO, United States). Stock solutions for these agents were prepared in dimethyl sulfoxide or Milli-Q water (Merck KGaA, Darmstadt, Germany), and were later diluted to the appropriate concentrations in either ECS. Except where indicated, all reagents were obtained from Sigma Chemical Co., (St. Louis, MO, United States).

### Measurement of the Intercellular Distance

Cells were imaged using the intensified charge-coupled device camera with microscope (Olympus and Hamamatsu Photonics). The distance from a mechanically stimulated MC to neighboring TGs was determined on the images by measuring the shortest distance between each pair of cell membranes with an analysis software (Hamamatsu Photonics).

### Statistical Analysis

Data were expressed as the means ± standard error (SE) of the mean of N observations, where N represents the number of separate experiments or cells. The Kruskal–Wallis test or Dunn’s *post hoc* test was used to determine non-parametric statistical significance. A *P* value of less than 0.05 was considered significant. Statistical analysis was performed using GraphPad Prism 5.0 (GraphPad Software, La Jolla, CA, United States).

## Results

### Mechanical Stimulation-Induced Ca^2+^ Influx in Quinacrine-Positive MCs

**FIGURE 1:**
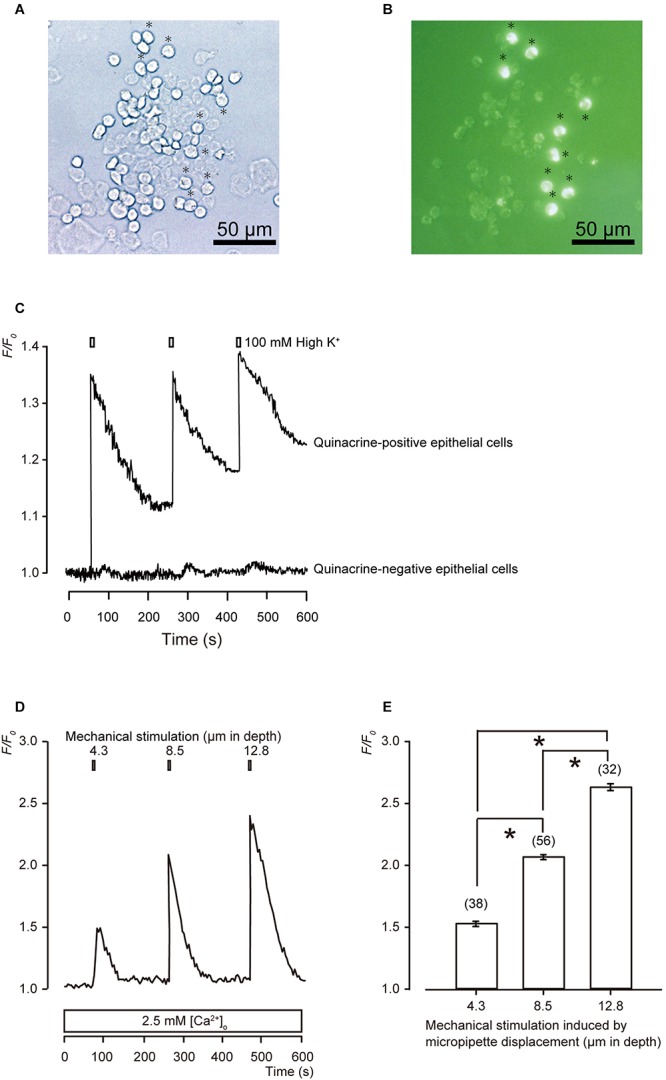
Morphological and functional characterizations of MCs. **(A,B)** Representative images of epithelial cells from hamster buccal mucosa by phase-contrast **(A)**, and quinacrine fluorescence imaging **(B)**. Quinacrine-positive epithelial cells are MCs (**B:** green). Scale bars are 50 μm for panels **(A,B)**. **(C)** Representative traces of [Ca^2+^]_i_ changes in the presence of 2.5 mM extracellular Ca^2+^ are shown, which are induced by the application of ECS containing high concentration of extracellular K^+^ (100 mM). High extracellular K^+^ solution induces membrane depolarization in both quinacrine-positive and -negative epithelial cells, and elicit [Ca^2+^]_i_ increase in quinacrine-positive cells, but not in quinacrine-negative cells. Resting value is shown as *F/F*_0_ = 1.0 in each trace. **(D)** Representative trace of transient increases in [Ca^2+^]_i_ during a series of mechanical stimulations, which is induced by vertical micropipette displacement downward by 4.3, 8.5, and 12.8 μm (upper boxes) in standard ECS. **(E)** Summary bar graph illustrates the *F/F*_0_ values as a function of vertical micropipette displacement. Each bar denotes the mean ± SE; numbers in parentheses correspond to the tested cells. ^*^P < 0.05.

In the bright-field images of acutely isolated epithelial cells (Figure 1A), we could not distinguish MCs from other epithelial cells visually. In the fluorescence images, we could distinguish MCs as quinacrine-positive cells (Figure 1B). To observe the functional characteristics of MCs, [Ca^2+^]_i_ in the fura-2-loaded primary cultured epithelial cells was measured by using ECS containing high extracellular K^+^ (100 mM) to induce membrane depolarization (Figure 1C). A series of applications of this ECS induced transient increases in [Ca^2+^]_i_ in all the quinacrine-positive cells (Figure 1C; *N* = 96), while other cells showed no responses (*N* = 23). The quinacrine-positive cells showing depolarization-induced [Ca^2+^]_i_ increase elicited by high K^+^ were identified as MCs (Tazaki and Suzuki, 1998).

When we applied mechanical stimulations to single MCs using a glass micropipette moving vertically in a downward direction from a position just above the surface (0 μm), significant transient increases in [Ca^2+^]_i_ were observed, corresponding to the mechanical stimulation strength of 4.3, 8.5, and 12.8 μm (Figures 1D,E).

**FIGURE 2:**
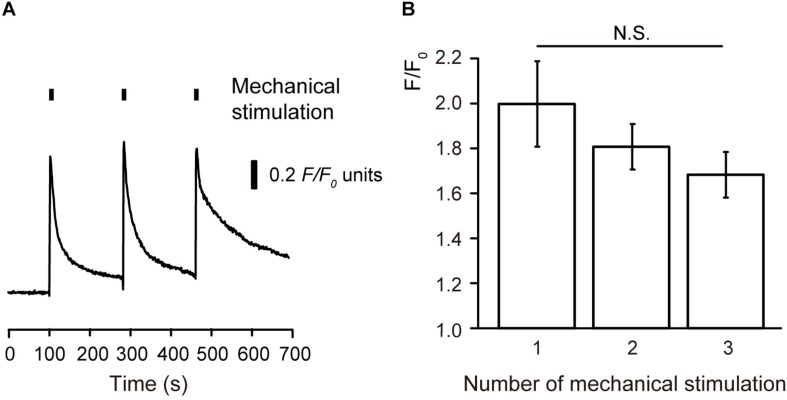
No desensitizing effects on the mechanical stimulation-induced [Ca^2+^]_i_ increase **(A)** Example trace of mechanical stimulation-induced [Ca^2+^]_i_ increase by repeated stimuli by micropipette displacement to a depth of 8.5 μm (black boxes) at a velocity of 2.8 μm/s. **(B)** Summary bar graph represents *F/F*_0_ values and shows no desensitizing effect after three repeated mechanical stimulations (*N* = 5). N.S., not significant among columns.

In addition, three repetitions of mechanical stimulation induced repeated transient [Ca^2+^]_i_ increases (Figure 2A). However, we could not observe any desensitizing effect on the [Ca^2+^]_i_ increases during the repeated mechanical stimulation (Figures 2A,B).

### Release of Diffusible Substances to the Extracellular Space Following Mechanical Stimulation to the MCs

**FIGURE 3:**
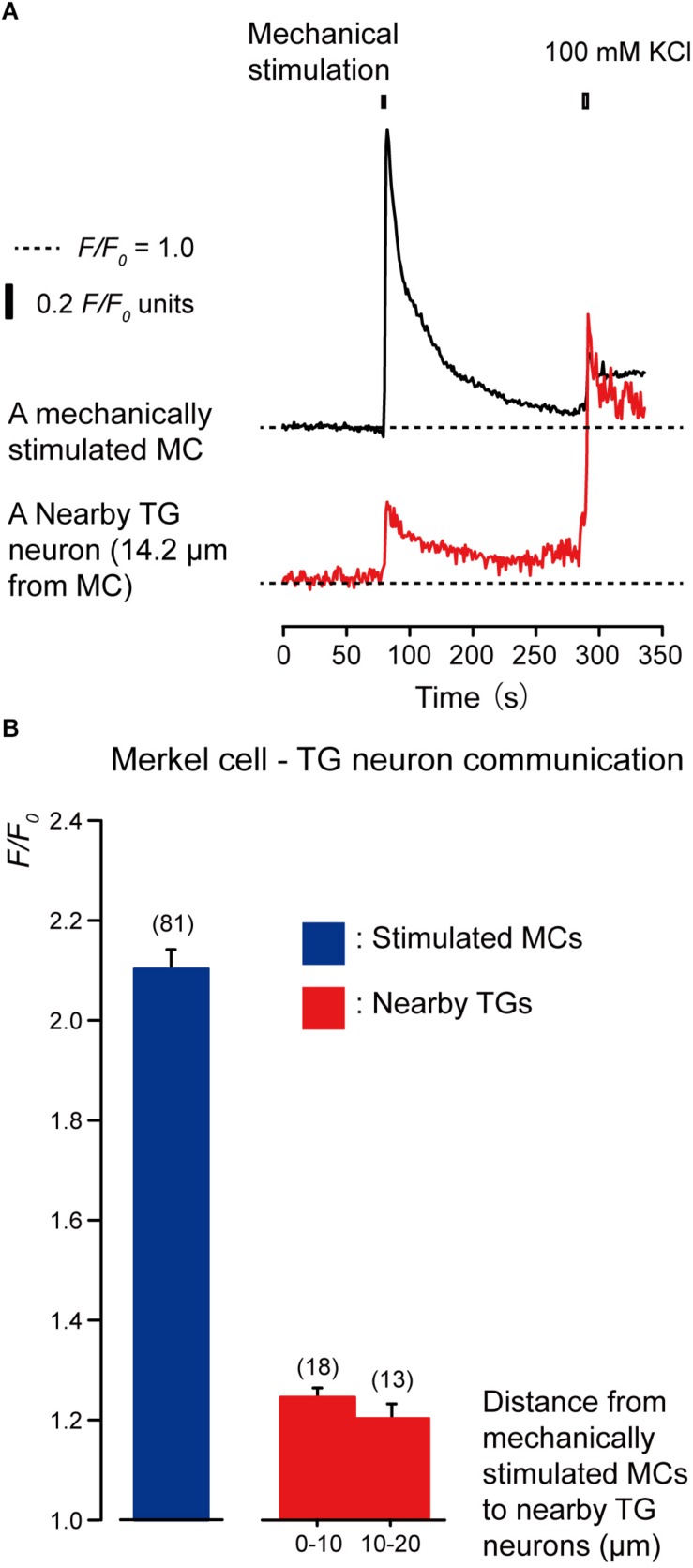
Merkel cells (MCs) are capable of releasing diffusible substances to the extracellular space following mechanical stimulation. **(A)** Representative traces of increases in [Ca^2+^]_i_ in the stimulated MC (black trace) and its nearby TGs (red trace). Horizontal dotted lines show the baseline (*F/F*_0_ = 1.0) for each response. Upper left filled box indicates the timing of mechanical stimulation by the displacement of a micropipette to a depth of 8.5 μm. Responses from the nearby TGs were recorded in cells at 14.2 μm from the stimulated MCs. The [Ca^2+^]_i_ increases by application of high concentration of extracellular K^+^ (100 mM; upper right box in panel **A**) to the quinacrine-positive MCs and TGs after each session of experiments could be observed. **(B)** The *F/F*_0_ values of the mechanically stimulated MCs (blue column) and nearby TGs (red columns) located within 0–10.0 μm and 10.1–20.0 μm from the stimulated MCs are shown. Bars represent the mean ± SE. Numbers in parentheses indicate the number of tested cells.

To investigate whether MCs are capable of establishing intercellular signaling with neurons by releasing neurotransmitters, we recorded [Ca^2+^]_i_ from cells in MC-TGs cocultures. The [Ca^2+^]_i_ was measured from mechanically stimulated MCs (i.e., quinacrine-positive epithelial cells) and several TGs located near the stimulated MCs. During the application of focal and direct mechanical stimulations to a single MCs (inducing cell deformation by displacement of a micropipette by 8.5 μm), we observed transient increases in [Ca^2+^]_i_ in the mechanically stimulated MCs (black in Figure 3A) to the peak value of 2.10 ± 0.04 *F/F*_0_ units (blue column in Figure 3B). In addition, we could also observe transient increases in [Ca^2+^]_i_ in the neighboring TGs (red in Figure 3A) during mechanical MCs stimulations. The peak values of [Ca^2+^]_i_ were 1.24 ± 0.03 and 1.20 ± 0.03 *F/F*_0_ units in TGs located within distances of 0–10.0 μm and 10.1–20.0 μm from mechanically stimulated MCs, respectively. Note that we applied high concentration of extracellular K^+^ (100 mM) to the quinacrine-positive cells and TGs after the completion of each session of experiments to discern that the recordings were acquired specifically from quinacrine-positive MCs, but not other epithelial cells, and from TGs, but not glial cells.

### ATP Release Following Mechanical Stimulation of MCs Is Unlikely

**FIGURE 4:**
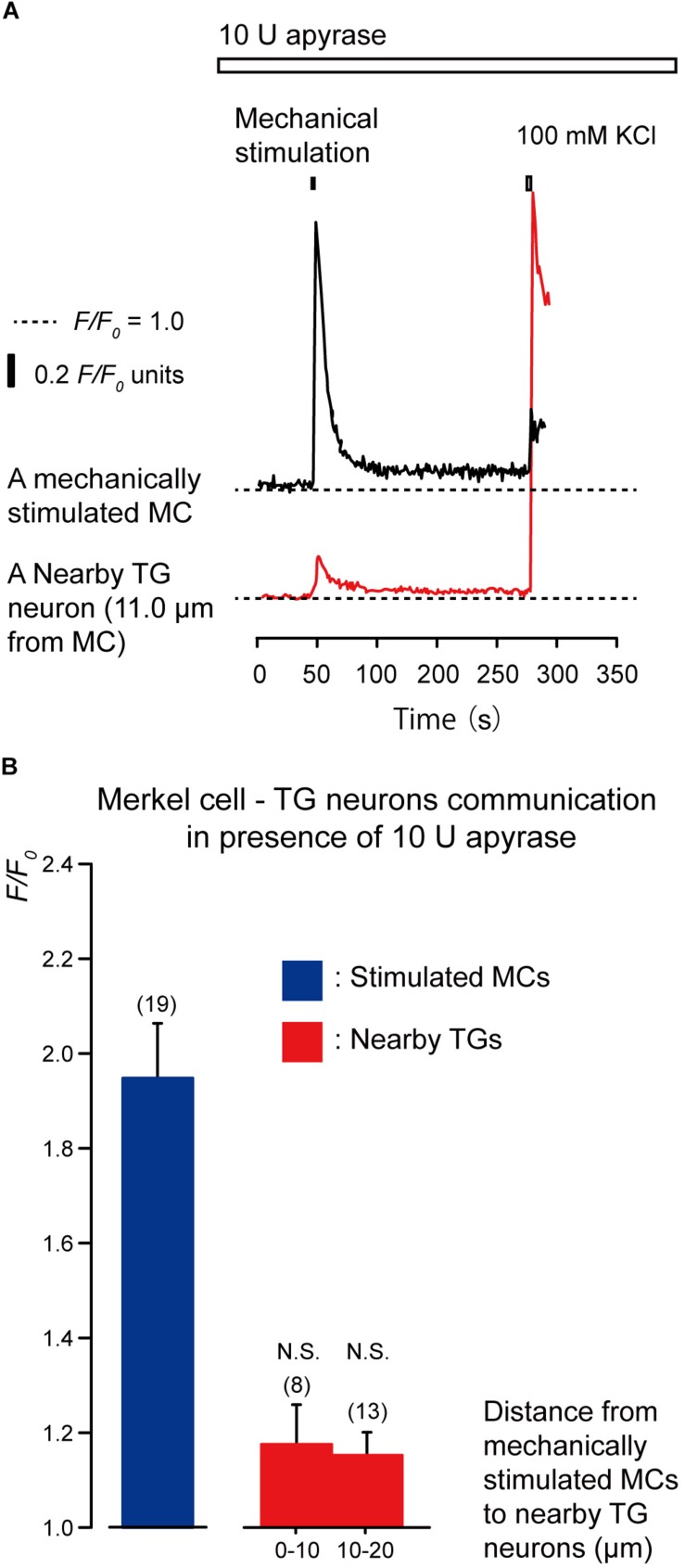
Effect of apyrase on [Ca^2+^]_i_ in neighboring TGs during stimulation of single MCs. **(A)** Typical traces of [Ca^2+^]_i_ increases in the stimulated MC (black trace) and its nearby TGs (red trace) in the presence of 10 U apyrase (upper white box). Horizontal dotted lines show the baseline (*F/F*_0_ = 1.0) for each response. Upper left filled box indicates the timing of mechanical stimulation by the displacement of a micropipette to a depth of 8.5 μm. Responses from the nearby TGs were recorded in cells at 11.0-μm from the stimulated MCs. The [Ca^2+^]_i_ increases by application of high concentration of extracellular K^+^ (100 mM; upper right box in panel **A**) to the quinacrine-positive MCs and TGs after each session of experiments could be observed. **(B)** The *F/F*_0_ values of the mechanically stimulated MCs (blue column) and nearby TGs (red columns) located within 0–10.0 μm and 10.1–20.0 μm from the stimulated MCs in the presence of 10 U apyrase are shown. Bars indicate the mean ± SE. Numbers in parentheses indicate the (tested cells. We could not observe any statistically significant differences between the *F/F*_0_ values of the mechanically stimulated MCs in the absence (Figure 3B) and presence of apyrase (*P* > 0.05). We could also not observe any statistically significant differences between the *F/F*_0_ values of the TGs located at 0–10.0 μm and 10.1–20.0 μm from the stimulated MCs in the absence (Figure 3B) and presence of apyrase (*P* > 0.05). N.S., not significant. Note that 10 U of apyrase will liberate 1.0 μmol of inorganic phosphate from ATP or ADP per minute, at pH 6.5 at 30°C, as ATPase.)

**FIGURE 5:**
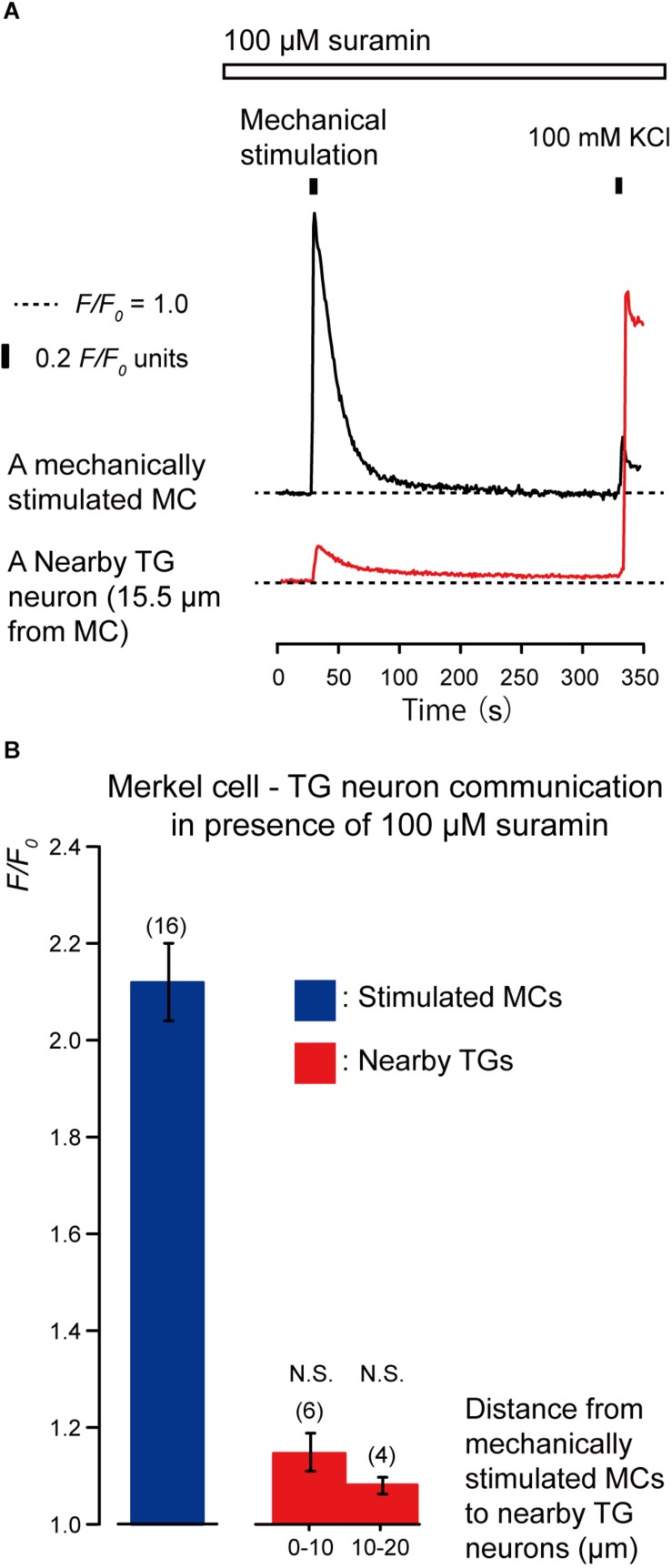
Effect of suramin on the [Ca^2+^]_i_ in MCs and nearby TGs during mechanical stimulation of a single MC. **(A)** Typical traces of [Ca^2+^]_i_ increases in the stimulated MC (black trace) and its nearby TGs (red trace) in the presence of 100 μM suramin (upper white box). Responses from the nearby TGs were recorded in cells at 15.5 μm from the stimulated MCs. The construction of the horizontal dotted lines, upper filled boxes, and [Ca^2+^]_i_ increases by application of high concentration of extracellular 100 mM K^+^ solution are the same as in Figure 4. **(B)** The *F/F*_0_ values of the mechanically stimulated MCs (blue column) and nearby TGs (red columns) located within 0–10.0 μm and 10.1–20.0 μm from the stimulated MCs in the presence of (100 μM suramin. Bars represent the mean ± SE. The numbers in parentheses indicate the tested cells. We could not observe any statistically significant differences between the *F/F*_0_ values of the mechanically stimulated MCs in the absence (Figure 3B) and presence of 100 μM suramin (*P* > 0.05). We could also not observe any statistically significant differences between the *F/F*_0_ values of the TGs located at 0–10.0 μm and 10.1–20.0 μm from the stimulated MCs in the absence (Figure 3B) and presence of suramin (*P* > 0.05). N.S., not significant.)

To clarify the substrate of neurotransmitters released by MCs following mechanical stimulation, we examined the effects of apyrase and suramin on [Ca^2+^]_i_ in stimulated MCs and neighboring TGs following mechanical stimulation of MCs. Apyrase is an extracellular ATP-degrading enzyme, while suramin is a non-selective P2 purinergic receptor antagonist. In the presence of 10 U of apyrase (in 1.5 mL of recording bath solution; standard ECS; Figure 4A) and 100 μM suramin (Figure 5A), the values of increase in [Ca^2+^]_i_ in both the mechanically stimulated MCs (black lines in Figures 4A, 5A) and neighboring TGs (red lines in Figures 4A, 5A) were not affected during direct mechanical stimulation (*P* > 0.05; Figures 4B, 5B), compared to those in the absence of apyrase and suramin in both cells (Figure 3).

### Glutamate Release From MCs via the Anion Channels That Activate Neuronal NMDA Receptors in TG Neurons Following Mechanical Stimulation of MCs

**FIGURE 6:**
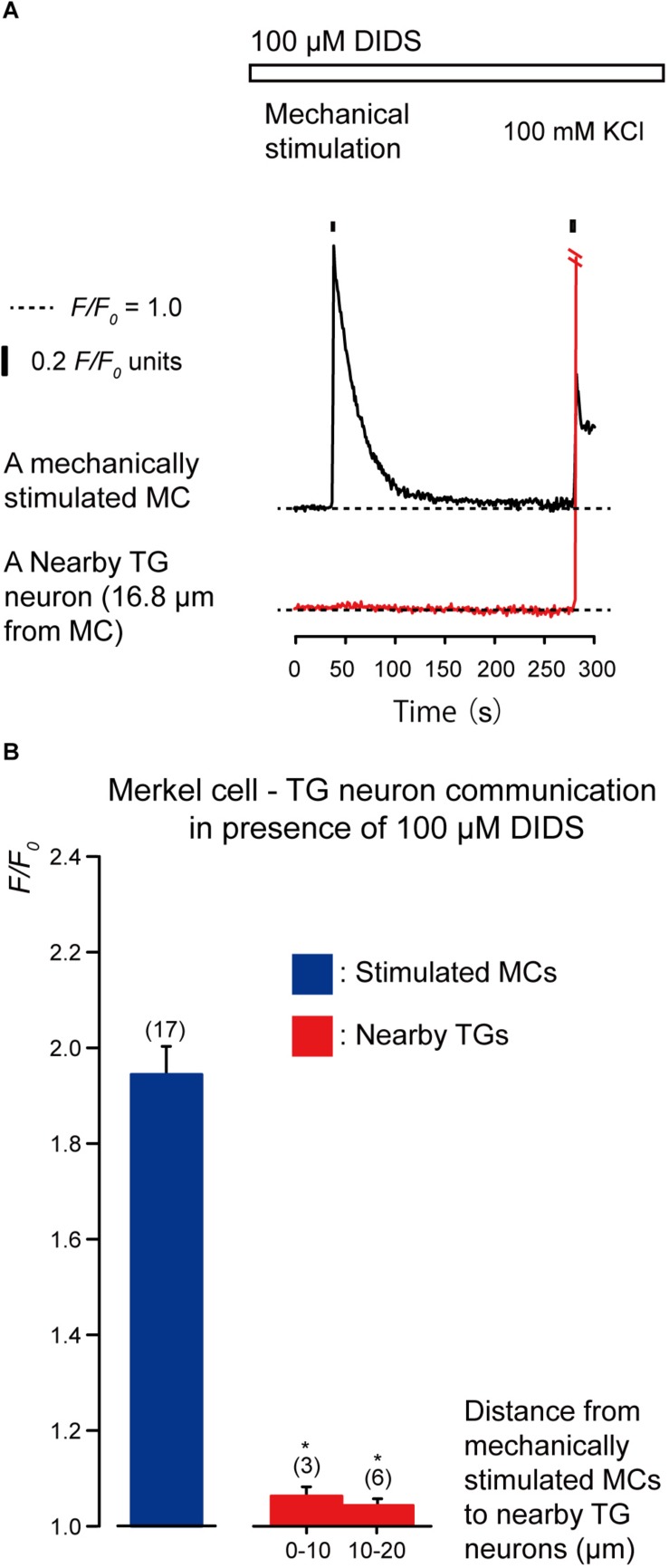
Effect of DIDS on the increases in [Ca^2+^]_i_ in stimulated MCs and nearby TGs during the mechanical stimulation of a single MC. **(A)** Traces of [Ca^2+^]_i_ increases in the stimulated MC (black trace) and its nearby TGs (red trace) in the presence of 100 μM DIDS (upper white box). Responses from the nearby TGs were recorded in cells at 16.8 μm from the stimulated MCs. The construction of the horizontal dotted lines, upper filled boxes and [Ca^2+^]_i_ increases by application of high concentration of extracellular 100 mM K^+^ solution are the same as in Figure 4. **(B)** The *F/F*_0_ values of the mechanically stimulated MCs (blue column) and nearby TGs (red columns) located within 0–10.0 μm and 10.1–20.0 μm of the mechanically stimulated MCs in the (presence of 100 μM DIDS. Bars represent the mean ± SE. The numbers in parentheses indicate the tested cells. We could not observe any statistically significant differences between the *F/F*_0_ values of the mechanically stimulated MCs in the absence (Figure 3B) and presence of 100 μM DIDS (*P* > 0.05). Statistically significant differences between the *F/F*_0_ values of TGs located at 0–10.0 μm and 10.1–20.0 μm from mechanically stimulated MCs in the absence (Figure 3B) and presence of 100 μM DIDS are observed. ^*^P < 0.05.)

**FIGURE 7:**
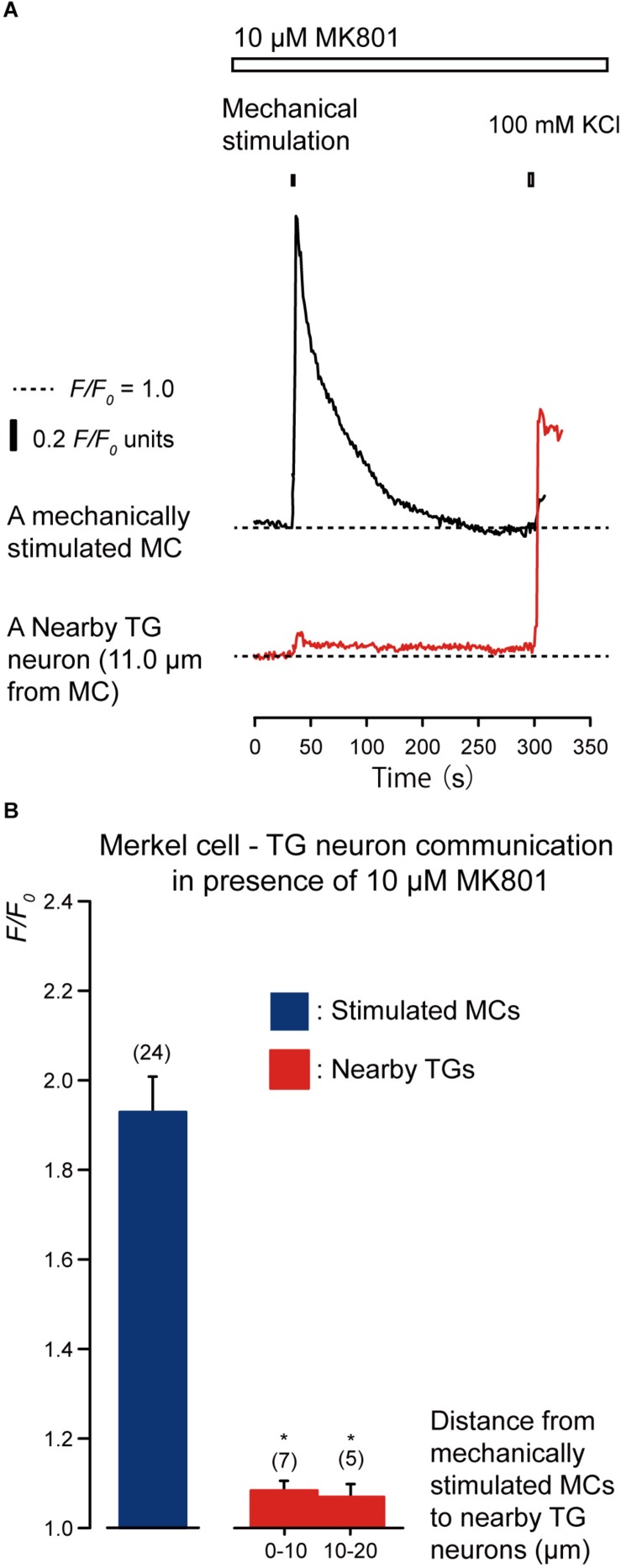
Effect of MK801 on the [Ca^2+^]_i_ in stimulated MCs and TGs close to the stimulated MCs following mechanical stimulation of a single MC. **(A)** Traces of [Ca^2+^]_i_ increases in the stimulated MC (black trace) and its nearby TGs (red trace) in the presence of 10 μM MK801 (upper white box). (Responses from nearby TGs were recorded in cells at 11.0 μm from the stimulated MCs. The construction of horizontal dotted lines, upper filled boxes, and [Ca^2+^]_i_ increases through the application of high concentration of extracellular 100 mM K^+^ solution are the same as in Figure 4. **(B)** In the presence of 10 μM MK801, the *F/F*_0_ value of the mechanically stimulated MCs (blue column) and nearby TGs (red columns); TGs located within 0–10.0 μm and 10.1–20.0 μm of the mechanically stimulated MCs are shown. Bars represent the mean ± SE. Numbers in parentheses indicate the tested cells. We could not observe any statistically significant differences between the *F/F*_0_ values of the mechanically stimulated MCs in the absence (Figure 3B) and presence of 10 μM MK801 (*P* > 0.05). Statistically significant differences between the *F/F*_0_ values of the TGs located at 0–10.0 μm and 10.1–20.0 μm from mechanically stimulated MCs in the absence (Figure 3B) and presence of MK801 (100 μM) ae observed. ^*^P < 0.05.)

We examined the effects of the antagonists of glutamate-permeable anion channels (100 μM DIDS) and non-competitive antagonist of the *N*-methyl-*D*-aspartate (NMDA) receptors (10 μM MK801) on the increase in [Ca^2+^]_i_ in both the stimulated MCs and neighboring TGs following the mechanical stimulation of a single MC (Figures 6, 7). The application of 100 μM DIDS or 10 μM MK801 significantly suppressed the [Ca^2+^]_i_ responses in the neighboring TGs (red lines) following MC mechanical stimulation (black lines; Figures 6A, 7A), compared to those obtained in the absence of DIDS and MK801 in both cells (Figure 3). In the presence of DIDS, the peak values of [Ca^2+^]_i_ in nearby TGs were 1.07 ± 0.02 and 1.05 ± 0.01 *F/F*_0_ units in TGs located within 0–10.0 μm and 10.1–20.0 μm of mechanically stimulated MCs, respectively, (Figure 6B). In the presence of MK801, the peak values of [Ca^2+^]_i_ in nearby TGs were 1.09 ± 0.02 and 1.07 ± 0.03 *F/F*_0_ units within 0–10.0 μm and 10.1–20.0 μm of mechanically stimulated MCs, respectively, (Figure 7B). Both in the presence of 100 μM DIDS or 10 μM MK801, the values of increase in [Ca^2+^]_i_ in the mechanically stimulated MCs (black lines in Figures 6A, 7A) were not affected (*P* > 0.05; blue columns in Figures 6B, 7B), compared to those value without DIDS and MK801 (Figure 3).

## Discussion

We obtained quinacrine-positive cells in the epithelial tissue from the touch domes of hamster buccal mucosa. The application of high-K^+^ solution resulted in a depolarization-induced [Ca^2+^]_i_ increase in single quinacrine-positive MCs; however, no increase in [Ca^2+^]_i_ was observed in quinacrine-negative epithelial cells. These results indicated that the quinacrine-positive epithelial cells used in this study showing [Ca^2+^]_i_ response have characteristics of MCs (Tazaki and Suzuki, 1998; Soya et al., 2014). Direct mechanical stimulation causing membrane deformation elicited a transmembrane [Ca^2+^]_i_ increase in MCs. We have previously reported the expression of mechanosensitive- and membrane stretch sensitive-TRP channels in MCs (Soya et al., 2014). In addition, the Piezo2 channels are essential as MC mechanical transducers (Woo et al., 2014). These results suggested that [Ca^2+^]_i_ increases induced by mechanical stimulation are mediated by mechanosensitive- and membrane stretch sensitive-TRP and/or Piezo2 channels. The [Ca^2+^]_i_ increase following mechanical stimulation observed in the present study showed no desensitizing effects during three repeated mechanical stimulations with 200 s intervals. Note that we applied the stimuli no more than three times to avoid unfavorable cell damage. Thus, the present results might not exclude the possibility of desensitization occurring from the fourth stimulation. In our previous study, however, five repeated applications of hypotonic ECS (200 mOsm/L) elicited repeated Ca^2+^ influx, but did not show a significant desensitizing effect on the influx (Soya et al., 2014; hypotonic ECS is capable of inducing plasma membrane stretch). Considering the present results together with our previous study (Soya et al., 2014), we suggested that the mechanosensors in MCs are capable of responding to repeated stimulations (Tsumura et al., 2012, 2013; Sato et al., 2015; Shibukawa et al., 2015; Nishiyama et al., 2016). However, further study will be needed to analyze the detailed adaptation properties (such as the time-dependent adaptive properties on the mechanical stimulus intervals or durations) of mechanical stimulation-induced responses in the MCs, as well as evoked-responses from the TGs following mechanical stimulation to the MCs.

Although we could not separate mixed populations of TGs into pure populations of A, peptidergic C, or non-peptidergic C neurons in the present coculture system, we could discern TGs by recording high-K^+^ solution-induced [Ca^2+^]_i_ increases. Thus, all the recordings were made from TGs, and not from any other glial cells (Kawaguchi et al., 2015a, b; Nishiyama et al., 2016).

We could observe [Ca^2+^]_i_ responses in the nearby TGs following mechanical stimulation to single MCs. These responses were inhibited by the application of NMDA-receptor antagonists, suggesting that the signaling molecule(s) released from single MCs diffused through the extracellular medium, and were capable of activating the NMDA receptors in TGs. In addition, extracellular DIDS suppressed the [Ca^2+^]_i_ increase in the neighboring TGs. Note that these antagonists (DIDS and MK801) did not affect the [Ca^2+^]_i_ increase in the mechanically stimulated MCs themselves. The putative pathway for the glutamate release is classified into two types: release via exocytotic vesicular transport and/or via transporters or channels (Malarkey and Parpura, 2008). Studies in mouse primary cultured astrocytes indicated that the DIDS sensitive-anion channel plays important roles as a glutamate transporter in the glutamate releasing pathway in cells (Liu et al., 2006; Sabirov and Okada, 2009). Gap-junction hemi-channels may also be involved in the glutamate release, but are insensitive to DIDS (Liu et al., 2009). Thus, the involvement of gap-junction hemi-channels in the glutamate release from MCs was unlikely. Vesicular glutamate transporters (VGLUTs) subtypes, including VGLUT1, VGLUT2, and VGLUT3, were expressed in MCs (Hitchcock et al., 2004; Nunzi et al., 2004). Although further studies will be needed to clarify the contribution of exocytotic vesicular transport and/or other transporters or channels in glutamate release, our results showed that DIDS-sensitive anion channels are involved in the glutamate release pathway in MCs. In addition, kynurenate, which is an antagonist of the ionotropic glutamate receptor, has been demonstrated to suppress the evoked responses of slowly adapting type I mechanoreceptor units in MC-rich sinus hair preparations (Fagan and Cahusac, 2001). Thus, the glutamate release is suggested to mediate most likely the mechanosensory transduction from MCs to nerve endings (Hitchcock et al., 2004; Nunzi et al., 2004). The inhibitory effects of DIDS and MK801 on the [Ca^2+^]_i_ increase in neighboring TGs suggested that glutamate is released into the extracellular space via glutamate releasing DIDS-sensitive anion channels (Liu et al., 2006, 2009; Malarkey and Parpura, 2008). Therefore, the results also implied that the glutamate released from MCs in response to cellular deformation may establish intercellular signal communication with neurons through the activation of NMDA receptors in neurons to mediate mechanosensory transduction in the MC-neurite complex.

In contrast, the extracellular ATP-degrading enzyme apyrase and the non-selective P2 purinergic receptor antagonist suramin did not affect the increases in [Ca^2+^]_i_ in both the mechanically stimulated MCs and the neighboring TGs. Recently, intercellular ATP signaling between taste receptor cells and neurons has been reported to generate taste signals (Murata et al., 2010; Taruno et al., 2013). In addition, neurotransmission from odontoblasts to neurons established by intercellular ATP signaling mediates sensory transduction sequence for the tooth pain (Sato et al., 2015; Shibukawa et al., 2015; Nishiyama et al., 2016). ATP has also been thought to be a candidate for neurotransmitter in the MC-neurite complex, since MCvesicles include ATP (Toyoshima and Shimamura, 1991). Therefore, we could not exclude the possibilities for the involvement of ATP as a neurotransmitter, and/or co-transmitter in the MC-neurite complexes. Further studies will be needed to explore other candidate neurotransmitters and their functional roles in the MC-neurite complex.

In conclusion, mechanical stimulation of MCs, which mimics membrane deformation, induced transmembrane [Ca^2+^]_i_ increases, which was followed by glutamate release, but not ATP, through glutamate-permeable anion channels. We suggest that MCs act as mechanoelectrical transducers, and are capable of releasing glutamate to the associated nerve endings in the MC-neurite complex. This neurotransmission may underlie the mechanosensory transduction induced by the MC-neurite complex. Ongoing experimental work is aimed at an electrophysiological approach using MC-TGs coculture to further study mechanoelectrical transduction mechanisms as well as synaptic transmissions between them. Recording mechanosensitive currents from MCs, as well as evoked-currents/potentials from the TGs following mechanical stimulation to the MCs, is also of immediate interest.

## Data Availability

No datasets were generated or analyzed for this study.

## Author Contributions

AH, MT, and YS designed the study. AH, MS, SO, WO, and MT acquired and analyzed the data. AH, MK, and YS interpreted the data. AH, MK, MT, and YS drafted the manuscript. All authors read and approved the final manuscript.

## Conflict of Interest Statement

The authors declare that the research was conducted in the absence of any commercial or financial relationships that could be construed as a potential conflict of interest.
